# Supplementary material for: Global patterns of the leaf economics spectrum in wetlands
Source: Nat Commun. 2020 Sep 9;11:4519. doi: 10.1038/s41467-020-18354-3 (PMC7481225; doi:10.1038/s41467-020-18354-3)
Supplement: Supplementary file 1 — Supplementary Information [file 41467_2020_18354_MOESM1_ESM.pdf]

Supplementary Information

Global patterns of the leaf economics spectrum in wetlands

Pan et al.

Supplementary Figure 1

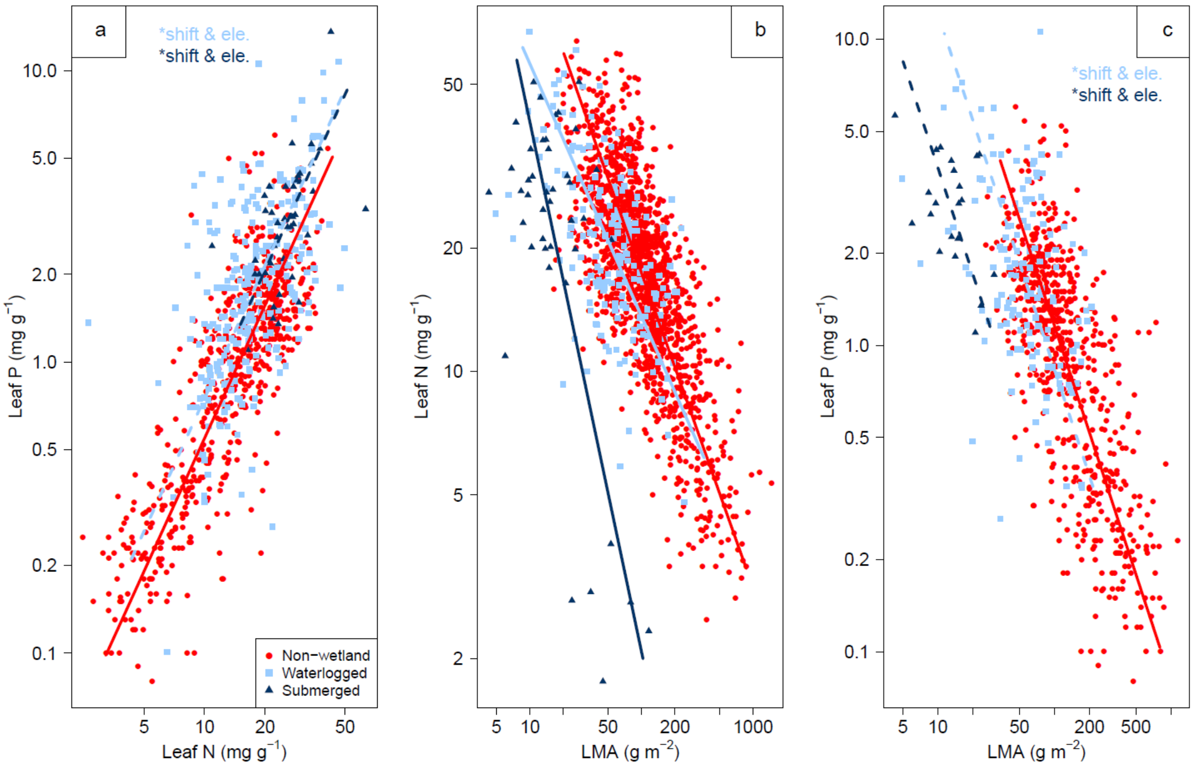

Supplementary Figure 1. The bivariate trait relationships between leaf P, N and LMA of the median value. **a** Leaf phosphorus (leaf P) vs leaf nitrogen (leaf N). **b** Leaf N vs. leaf dry mass per unit area (LMA). **c** Leaf P vs. LMA. The waterlogged and submerged wetland plants are shown in light blue squares and dark blue triangles, respectively. The non-wetland plant data from GLOPNET [1] are shown in red circles with a solid red line. If the slope for wetland plants differs significantly from that of non-wetland plants, this is indicated by a solid dark or light blue line, for waterlogged and submerged plants, respectively. Dashed lines with the notation of **shift* and/or **ele.* identify a significant shift along the common slope, and/or significant elevation differences among parallel slopes, respectively. Note that graph axes are log_{10} scaled.

Supplementary Figure 2

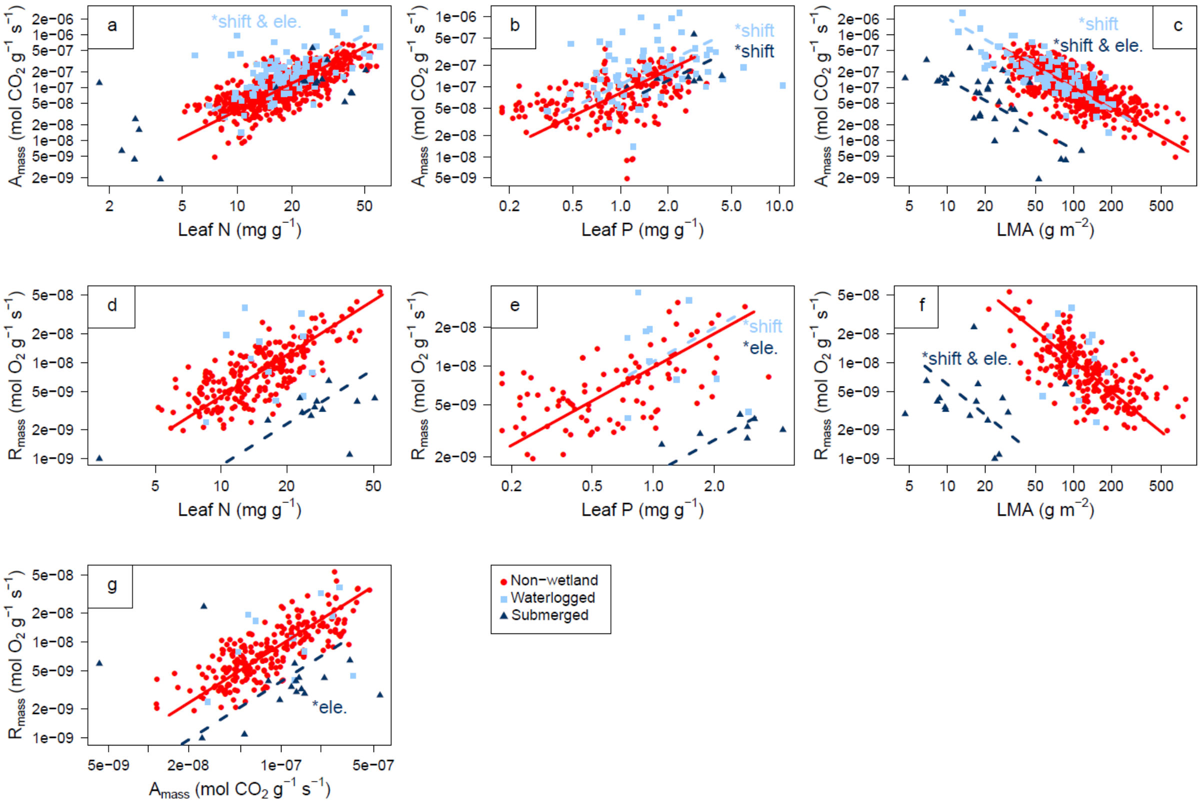

Supplementary Figure 2. The bivariate relationships with photosynthetic rate and dark respiration rate of the median value. **a** Photosynthetic rate (A_{mass}) vs. leaf nitrogen (leaf N). **b** A_{mass} vs. leaf phosphorus (leaf P). **c** A_{mass} vs. leaf dry mass per unit area (LMA). **d** Dark respiration rate (R_{mass}) vs. leaf N. **e** R_{mass} vs. leaf P. **f** R_{mass} vs. LMA. **g** A_{mass} vs. R_{mass}. The waterlogged and submerged wetland plants are shown in light blue squares and dark blue triangles, respectively. The non-wetland plant data from GLOPNET [1] are shown in red circles with a solid red line. If the slope for wetland plants differs significantly from that of non-wetland plants, this is indicated by a solid dark or light blue line, for waterlogged and submerged plants, respectively. Dashed lines with the notation of **shift* and/or **ele.* identify a significant shift along the common slope, and/or significant elevation differences among parallel slopes, respectively. Note that graph axes are $\log_{10}$ scaled.

Supplementary Figure 3

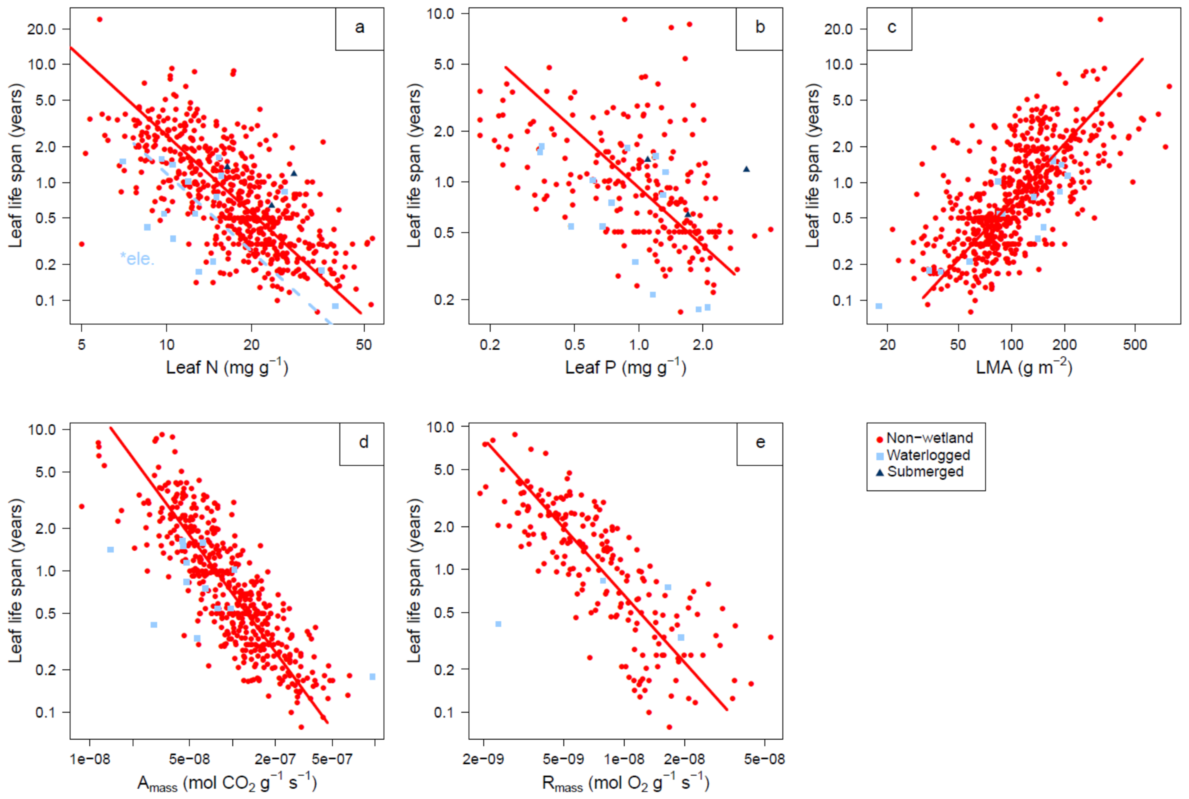

Supplementary Figure 3. The bivariate relationships with leaf life span of the median value.

a Leaf life span vs. leaf nitrogen (leaf N). **b** Leaf life span vs. leaf phosphorus (leaf P). **c** Leaf life span vs. leaf dry mass per unit area (LMA). **d** Leaf life span vs. photosynthetic rate (A_{mass}). **e** Leaf life span vs. dark respiration rate (R_{mass}). The waterlogged and submerged wetland plants are shown in light blue squares and dark blue triangles, respectively. The non-wetland plant data from GLOPNET [1] are shown in red circles with a solid red line. The dashed light blue line with the notation of **ele.* identifies significant elevation differences among parallel slopes respectively. Note that graph axes are $\log_{10}$ scaled and the absence of leaf life span data coupled to LMA, A_{mass}, or R_{mass} for submerged plants.

Supplementary Figure 4

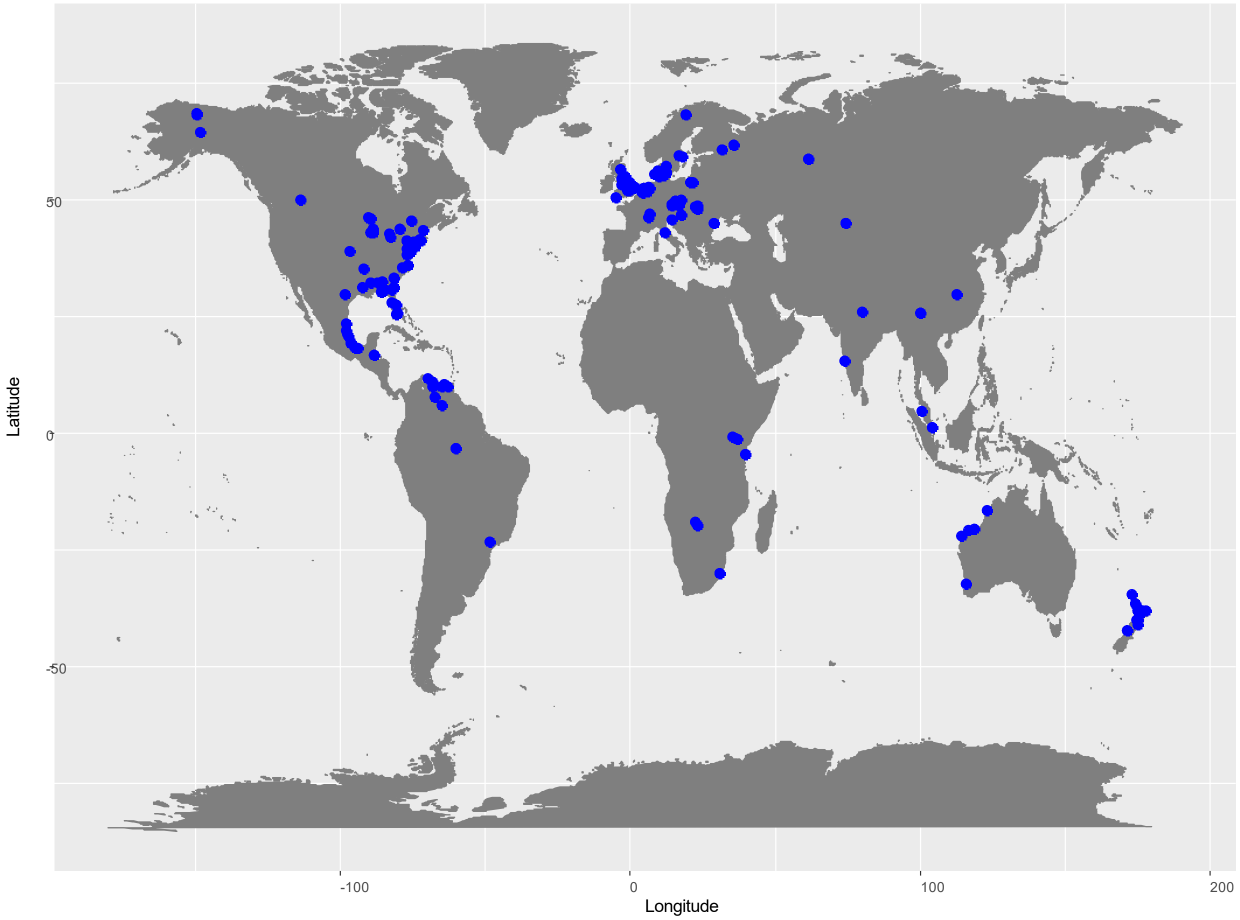

Supplementary Figure 4. Map of the sampling sites for wetland plants from which accurate spatial location information was available (933 out of 2789 records).

Supplementary Figure 5

PRISMA 2009 Flow Diagram

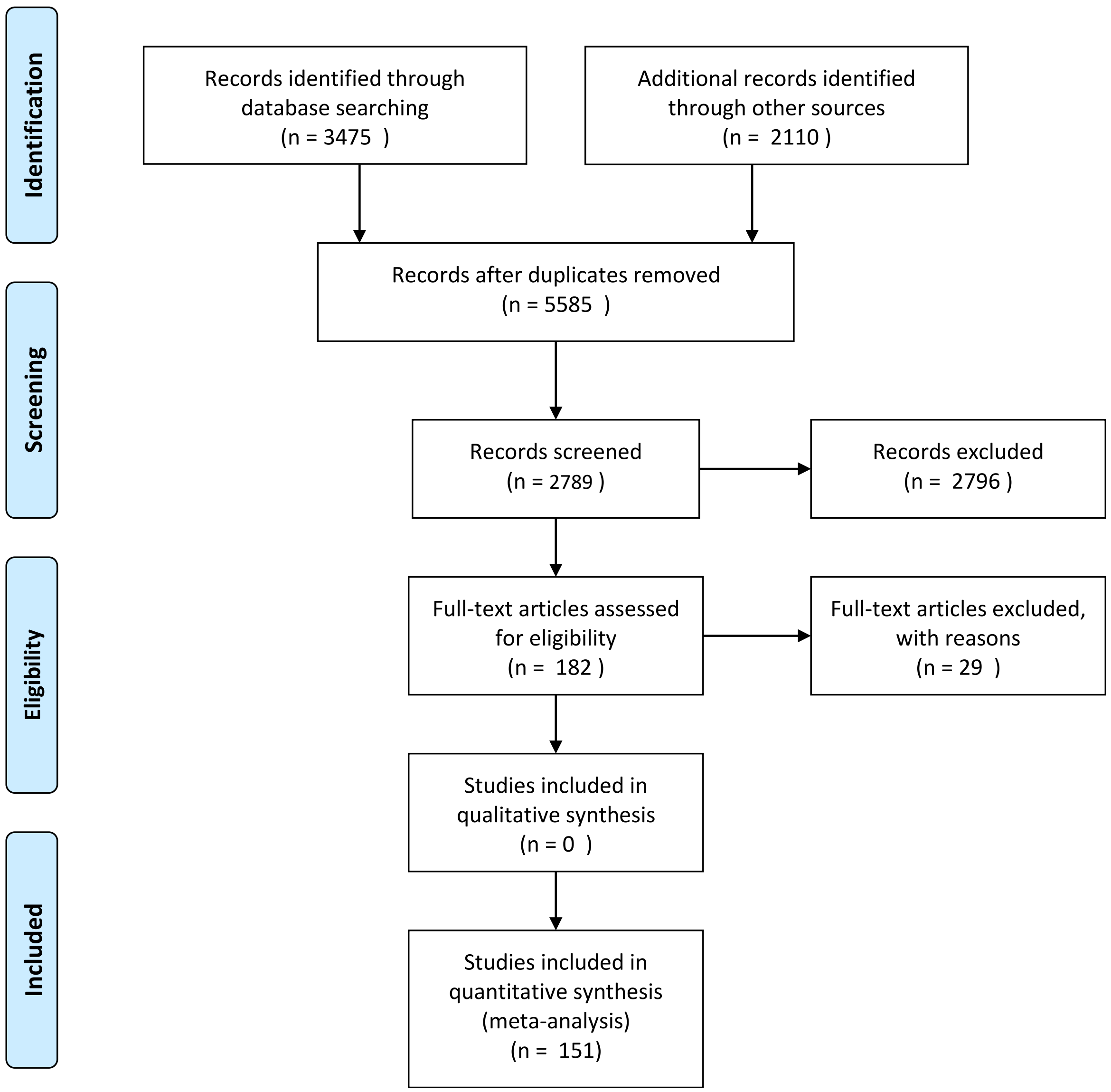

From: Moher D, Liberati A, Tetzlaff J, Altman DG, The PRISMA Group (2009). *P*referred *R*eporting *I*tems for *S*ystematic Reviews and *M*eta-*A*nalyses: The PRISMA Statement. PLoS Med 6(7): e1000097. doi:10.1371/journal.pmed1000097

For more information, visit www.prisma-statement.org.

Supplementary Figure 5. The PRISMA flowchart statement.

Supplementary Table 1

Supplementary Table 1. The confidence intervals (CIs) of R^2 at the 95% significance level.

	log LMA	log N_{mass}	log P_{mass}	log A_{mass}	log R_{mass}	Plant type
log N_{mass}	0.33* (0.22, 0.44)					Waterlogged
	(P<0.001; n=178)					
	0.23* (0.02, 0.44)					Submerged
	(P=0.001; n=42)					
	0.57* (0.54, 0.60)					Non-wetland
	(P<0.001;n=1322)					
log P_{mass}	0.17* (0.06, 0.28)	0.31* (0.22, 0.40)				Waterlogged
	(P<0.001; n=135)	(P<0.001; n=264)				
	0.12 (-0.12, 0.36)	0.31* (0.09, 0.53)				Submerged
	(P=0.123; n=21)	(P<0.001; n=41)				
	0.52* (0.46, 0.58)	0.70* (0.66, 0.74)				Non-wetland
	(P<0.001; n=561)	(P<0.001; n=555)				
log A_{mass}	0.59* (0.46, 0.72)	0.27* (0.12, 0.42)	0.12* (-0.02, 0.26)			Waterlogged
	(P<0.001; n=91)	(P<0.001; n=90)	(P=0.003; n=72)			
	0.56* (0.34, 0.78)	0.49* (0.20, 0.78)	0.11(-0.22, 0.44)			Submerged
	(P<0.001; n=31)	(P=0.001; n=18)	(P=0.468; n=7)			
	0.51* (0.45, 0.57)	0.54* (0.48, 0.60)	0.19* (0.09, 0.29)			Non-wetland
	(P<0.001; n=579)	(P<0.001; n=537)	(P<0.001; n=171)			
log R_{mass}	0.03 (-0.13, 0.19)	0.02 (-0.12, 0.16)	0.13 (-0.19, 0.45)	0.14 (-0.17, 0.45)		Waterlogged
	(P=0.626; n=11)	(P=0.681; n=11)	(P=0.307; n=10)	(P=0.255; n=11)		
	0.00 (-0.07, 0.09)	0.27 (-0.09, 0.63)	0.13 (-0.22, 0.48)	0.05 (-0.13, 0.23)		Submerged
	(P=0.895; n=16)	(P=0.083; n=12)	(P=0.423; n=7)	(P=0.383; n=16)		
	0.45* (0.36, 0.54)	0.58* (0.50, 0.66)	0.37* (0.21, 0.53)	0.61* (0.53, 0.69)		Non-wetland
	(P<0.001; n=228)	(P<0.001; n=221)	(P<0.001; n=84)	(P<0.001; n=220)		
log LL	0.78* (0.61, 0.95)	0.35 (0.02, 0.68)	0.34 (-0.01, 0.69)	0.40 (0.05, 0.75)	0.00 (-0.11, 0.13)	Waterlogged
	(P<0.001; n=16)	(P=0.015; n=16)	(P=0.028; n=14)	(P=0.021; n=13)	(P=0.960; n=4)	
	---	0.01 (-0.11, 0.13)	0.02 (-0.15, 0.19)	---	---	Submerged
	---	(P=0.949; n=3)	(P=0.917; n=3)	---	---	
	0.43* (0.36, 0.50)	0.45* (0.38, 0.52)	0.27* (0.16, 0.38)	0.69* (0.64, 0.74)	0.62* (0.54, 0.70)	Non-wetland
	(P<0.001; n=503)	(P<0.001; n=489)	(P<0.001; n=173)	(P<0.001; n=382)	(P<0.001; n=187)	

Note: The R^2 values are given in the first row with their confidence intervals (CIs) at the 95% significance level in brackets. The second row indicates the P value of the slope and sample size n. A bright green indicates that no overlap among the R^2 CIs range between waterlogged and non-wetland plants; the bright blue indicates no overlap among the R^2 CIs range between submerged and non-wetland plants.

Supplementary Table 2

Supplementary Table 2. Difference in slopes between wetland plants and non-wetland plants.

		log LMA	log N_{mass}	log P_{mass}	log A_{mass}	log R_{mass}
log N_{mass}	Wat.	**SlopD=0.18; n=178; P< 0.001**				
	Sub.	**SlopD=-0.52; n=42; P< 0.001**				
log P_{mass}	Wat.	SlopD=0.19; n=135; P=0.058	SlopD=0.09; n=264; P=0.274			
	Sub.	SlopD=0.45; n=21; P=0.039	SlopD=0.02; n=41; P=0.896			
log A_{mass}	Wat.	SlopD=-0.03; n=91; P=0.780	SlopD=0.34; n=90; P=0.089	SlopD=0.21; n=72; P=0.137		
	Sub.	**SlopD=-0.68; n=31; P=0.002**	SlopD=-0.37; n=18; P=0.149	SlopD=0.53; n=7; P=0.357		
log R_{mass}	Wat.	SlopD=-1.2; n=11; P=0.032	SlopD=0.92; n=11; P=0.159	SlopD=-0.65; n=10; P=0.105	SlopD=0.14; n=11; P=0.588	
	Sub.	SlopD=1.88; n=16; P=0.429	**SlopD=-0.85; n=12; P=0.004**	SlopD=0.02; n=7; P=0.900	SlopD=-1.38; n=16; P=0.030	
log LL	Wat.	SlopD=-0.35; n=16; P=0.052	SlopD=0.21; n=16; P=0.498	SlopD=-0.31; n=14; P=0.562	SlopD=0.64; n=13; P=0.011	SlopD=2.04; n=4; P=0.217
	Sub.	n.d.	SlopD=0.4; n=3; P=0.854	SlopD=0.09; n=3; P=0.874	n.d.	n.d.

Note: In each cell, the difference in slopes (SlopD) between wetland and non-wetland plants, sample size n and P value are provided. Significant differences are in bold ($P<0.01$). Slope differences bigger than 1 or less than -1 are labelled in red colour. Several combinations of leaf life span and other traits at submerged conditions had too few observations to be analysed (indicated as n.d.= not determined).

Supplementary Table 3

Supplementary Table 3. Bivariate relationships between leaf economics traits based on the median value.

	log LMA	log N_{mass}	log P_{mass}	log A_{mass}	log R_{mass}	log LL	Plant Type
log LMA		-0.62 (-0.70, -0.54)	-1.00 (-1.17, -0.86)	-1.34 (-1.53, -1.71)	-2.33 (-4.62, -1.18)	1.25 (0.95, 1.65)	Waterlogged
		-1.29 (-1.66, -1.00)	-0.88 (-1.34, -0.58)	-1.85 (-2.40, -1.42)	-1.01 (-1.74, -0.59)	--- --- ---	Submerged
		-0.77 (-0.79, -0.74)	-1.19 (-1.25, -1.13)	-1.31 (-1.38, -1.24)	-1.04 (-1.14, -0.95)	1.66 (1.56, 1.76)	Non-wetland
log N_{mass}	0.31*		1.58 (1.43, 1.75)	2.00 (1.66, 2.40)	2.40 (1.20, 4.80)	-1.88 (-2.98, -1.18)	Waterlogged
	0.36*		1.64 (1.25, 2.14)	1.26 (0.87, 1.83)	0.73 (0.43, 1.26)	-1.46 (-34.72, -0.06)	Submerged
	0.57*		1.50 (1.44, 1.57)	1.70 (1.62, 1.80)	1.44 (1.33, 1.57)	-2.20 (-2.34, -2.07)	Non-wetland
log P_{mass}	0.16*	0.32*		1.28 (1.02, 1.60)	-1.68 (-3.38, -0.83)	-1.44 (-2.34, -0.89)	Waterlogged
	0.20	0.30*		1.28 (0.50, 3.28)	0.41 (0.18, 0.96)	-0.75 (-19.08, -0.03)	Submerged
	0.52*	0.71*		1.04 (0.93, 1.17)	0.86 (0.72, 1.01)	-1.13 (-1.27, -1.01)	Non-wetland
log A_{mass}	0.59*	0.24*	0.10*		1.09 (0.57, 2.09)	-0.71 (-1.16, -0.43)	Waterlogged
	0.51*	0.49*	0.10		-0.63 (-1.08, -0.37)	--- --- ---	Submerged
	0.50*	0.54*	0.19*		0.87 (0.81, 0.95)	-1.38 (-1.45, -1.31)	Non-wetland
log R_{mass}	0.04	0.02	0.13	0.14		0.47 (0.08, 2.90)	Waterlogged
	0.01	0.35	0.33	0.01		--- --- ---	Submerged
	0.46*	0.58*	0.33*	0.61*		-1.58 (-1.72, -1.45)	Non-wetland
log LL	0.77*	0.29	0.35	0.40	0.01		Waterlogged
	---	0.12	0.00	---	---		Submerged
	0.45*	0.46*	0.24*	0.69*	0.63*		Non-wetland

Note: The bivariate relationships between including leaf life span (LL), leaf dry mass per unit area (LMA), photosynthetic rate (A_{mass}), leaf nitrogen (leaf N, wt/wt), leaf phosphorus (leaf P,wt/wt), dark respiration rate (R_{mass}) are provided for wetland plants and for comparison given for non-wetland plants. Standardized major axis (SMA) slopes with 95% confidence interval are given in the upper-right section of the table (x variable in column 1, y variable in row 1); coefficients of determination (R^2) of SMA and sample sizes are given in the lower-left section of the matrix. The different rows identify statistical properties calculated for waterlogged and submerged wetland plants, and for non-wetland species from the GLOPNET database [1], respectively. The asterisk indicates significant correlation at P<0.01.

Supplementary Table 4

Supplementary Table 4. Comparison of the bivariate relationships in wetland vs. non-wetland plants based on the median value.

	log LMA			log N_{mass}			log P_{mass}			log A_{mass}			log R_{mass}		
log N_{mass}															
log P_{mass}															
log A_{mass}															
log R_{mass}															
log LL															
	---	----	---							---	---	---	---	---	---

Note: The differences in slopes (first column), shift along the common slope (second column) and elevation differences among parallel slopes (third column) between non-wetland plants vs. waterlogged wetland plants (first row) and vs. submerged wetland plants (second row), respectively, were analyzed by SMA. Significant differences are in black (P<0.01), non-significant differences in light grey (P>0.01). If slopes are significantly different, this implies differences both in the direction and location of the relationship in trait space [2]. In those conditions, the shift along the common slope and the elevation difference among parallel slopes cannot be tested [2] (and shown in dark grey).

Supplementary Methods

When evaluating plants' performance along a gradient from dry to wet conditions, the Ellenberg moisture indicator is a useful summary of the plant general adaptation to habitat wetness [3]. It effectively represents the synergy of the adaptation to the complex adverse wetland conditions (the wet end of the gradient) and the suite of adaptation traits needed to cope with those conditions. The Ellenberg moisture indicator classification consists of 12 levels corresponding to prevalence along a wetness gradient from 1 (very dry) to 12 (aquatic) [3]. Wetland plants usually occupy the higher range from level 4 [4] up to level 12 containing obligate aquatic plants. Studies have shown that the Ellenberg moisture indicator is associated to plant functional traits and soil variables [4–6].

In this study, the Ellenberg moisture indicator was obtained from both the European mainland [3] and the British vegetation descriptions [7]. Moreover, to make the Ellenberg moisture indicator applicable for a global analysis, we related the Ellenberg moisture indicator values with the USDA wetland plant classification as proposed by Lichvar et al. 2016 (http://wetland-plants.usace.army.mil/). This system principally categorizes 8092 plant species occurring in the United States of America into five wetness indicator categories. The categories include sequentially Obligate (OBL) species with 99% occurrence in wetlands, Facultative Wetland (FACW) with 67%-99% occurrence in wetlands, Facultative (FAC) with 34%-66% occurrence in wetlands, Facultative Upland (FACU) with 1%-33% occurrence in wetlands, and Upland (UPL) with less than 1% occurrence in wetlands [8]. We coded the five USDA indicator categories from UPL to OBL into 1-5 ordinal classes and refer to this indicator system as the USDA indicator. All species selected in the analysis had a Ellenberg moisture or a USDA indicator value.

Using a simple linear regression of the Ellenberg moisture and USDA indicator for the 328 plants common to both datasets, we were able to convert USDA indicators to Ellenberg values for all remaining species using the following relationship:

*Ellenberg moisture indicator= 1.6531*USDA indicator+1.5084 (R^2=0.744, n=328)*

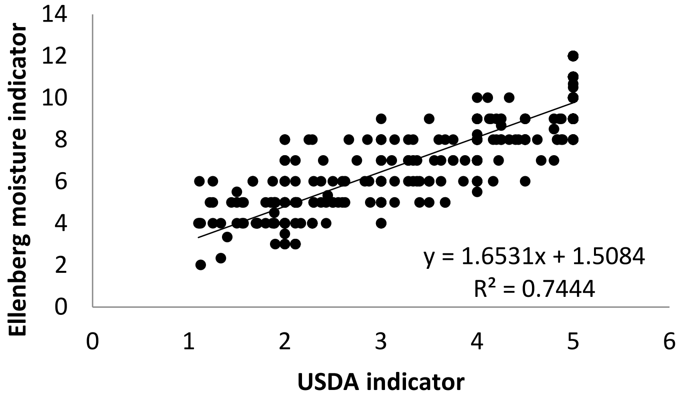

The joint Ellenberg moisture indicator value estimates were applied for the analyses presented in this paper.

References

1. Wright, I. J. *et al.* The worldwide leaf economics spectrum. *Nature* **428**, 821–827 (2004).
2. Warton, D. I., Wright, I. J., Falster, D. S. & Westoby, M. Bivariate line-fitting methods for allometry. *Biol. Rev. Camb. Philos. Soc.* **81**, 259–291 (2006).
3. Ellenberg, H. H. *Vegetation ecology of central Europe*. (Cambridge University Press, 1988).
4. Shipley, B. *et al.* Predicting habitat affinities of plant species using commonly measured functional traits. *J. Veg. Sci.* **28**, 1082–1095 (2017).
5. Bartelheimer, M. & Poschlod, P. Functional characterizations of Ellenberg indicator values - a review on ecophysiological determinants. *Funct. Ecol.* **30**, 506–516 (2016).
6. Bartholomeus, R. P., Witte, J. M., van Bodegom, P. M. & Aerts, R. The need of data harmonization to derive robust empirical relationships between soil conditions and vegetation. *J. Veg. Sci.* **19**, 799–808 (2008).
7. Hill, M. O., Roy, D. B., Mountford, J. O. & Bunce, R. G. H. Extending Ellenberg's indicator values to a new area: An algorithmic approach. *J. Appl. Ecol.* **37**, 3–15 (2000).
8. Lichvar, R. W., Banks, D. L., Kirchner, W. N. & Melvin, N. C. The National Wetland Plant List: 2016 wetland ratings. *Phytoneuron* **30**, 1–7 (2016).
