# Supplementary material for: Global patterns of the leaf economics spectrum in wetlands
Source: Nat Commun. 2020 Sep 9;11:4519. doi: 10.1038/s41467-020-18354-3 (PMC7481225; doi:10.1038/s41467-020-18354-3)
Supplement: Supplementary file 3 — Reporting Summary [file 41467_2020_18354_MOESM3_ESM.pdf]

nature research

Corresponding author(s): Yingji Pan

Last updated by author(s): Jul 22, 2020

Reporting Summary

Nature Research wishes to improve the reproducibility of the work that we publish. This form provides structure for consistency and transparency in reporting. For further information on Nature Research policies, see our Editorial Policies and the Editorial Policy Checklist.

Statistics

For all statistical analyses, confirm that the following items are present in the figure legend, table legend, main text, or Methods section.

n/a	Confirmed	
☐	☒	The exact sample size (n) for each experimental group/condition, given as a discrete number and unit of measurement
☐	☒	A statement on whether measurements were taken from distinct samples or whether the same sample was measured repeatedly
☐	☒	The statistical test(s) used AND whether they are one- or two-sided<br>*Only common tests should be described solely by name; describe more complex techniques in the Methods section.*
☐	☒	A description of all covariates tested
☐	☒	A description of any assumptions or corrections, such as tests of normality and adjustment for multiple comparisons
☐	☒	A full description of the statistical parameters including central tendency (e.g. means) or other basic estimates (e.g. regression coefficient) AND variation (e.g. standard deviation) or associated estimates of uncertainty (e.g. confidence intervals)
☐	☒	For null hypothesis testing, the test statistic (e.g. F, t, r) with confidence intervals, effect sizes, degrees of freedom and P value noted<br>*Give P values as exact values whenever suitable.*
☒	☐	For Bayesian analysis, information on the choice of priors and Markov chain Monte Carlo settings
☒	☐	For hierarchical and complex designs, identification of the appropriate level for tests and full reporting of outcomes
☐	☒	Estimates of effect sizes (e.g. Cohen's d, Pearson's r), indicating how they were calculated

Our web collection on statistics for biologists contains articles on many of the points above.

Software and code

Policy information about availability of computer code

Data collection	No software was used for data collection.
Data analysis	All data was analysed through open source packages in R software (3.6.0). All packages including their versions have been described in the manuscript with reference to the relevant statistical analysis.

For manuscripts utilizing custom algorithms or software that are central to the research but not yet described in published literature, software must be made available to editors and reviewers. We strongly encourage code deposition in a community repository (e.g. GitHub). See the Nature Research guidelines for submitting code & software for further information.

Data

Policy information about availability of data

All manuscripts must include a data availability statement. This statement should provide the following information, where applicable:

- Accession codes, unique identifiers, or web links for publicly available datasets
- A list of figures that have associated raw data
- A description of any restrictions on data availability

The plant trait data that support the findings of this study and underlie its figures and tables can be downloaded from DRYAD repository (https://doi.org/10.5061/dryad.v6wwpzgsq). The GLOPNET data from which our non-terrestrial data were derived are available from: https://www.nature.com/articles/nature02403#Sec15.

Field-specific reporting

Please select the one below that is the best fit for your research. If you are not sure, read the appropriate sections before making your selection.

☐ Life sciences ☐ Behavioural & social sciences ☒ Ecological, evolutionary & environmental sciences

For a reference copy of the document with all sections, see nature.com/documents/nr-reporting-summary-flat.pdf

Ecological, evolutionary & environmental sciences study design

All studies must disclose on these points even when the disclosure is negative.

Study description	The study is a meta-analysis of literature data. We designed a database in which for a given species observations on traits were compiled. For each species record, we thus noted the species, study system, environmental conditions (if available), and for each trait average, sample size, standard deviation and unit. We also documented the reference from which the record had been derived. We only compiled data for species normally occurring in wetlands, or measured at wetland conditions.
Research sample	We defined wetland plants as species that mainly occur in (or are exposed to) wetland habitats as described by the Ramsar Convention. Traits of wetland plants were collected from scientific literature (and supplemented with a few unpublished data sources from field observations of our co-authors). The population sampled is meant to represent the entire globe, even though data limitations may put constraints on this representation. Those constraints are discussed in the manuscript. We did not include data from other trait databases that are dominated by terrestrial records, including TRY, because the few records available for wetland plants in these databases do not have a sufficiently detailed habitat description that would allow the differentiation between waterlogged and submerged required for our analysis. The GLOPNET data (https://www.nature.com/articles/nature02403#Sec15) were obtained to represent non-wetland plants as a background reference of trait values.
Sampling strategy	We collected leaf economics traits for wetland plants on a global scale including those individuals exposed to intermittent/permanent wetland conditions (waterlogged or flooded) from both field and experiment measurements. The wetland plant leaf economics trait dataset was compiled based on a systematic search in Web of Science and Google Scholar (last updated on the 5th June 2018). The literature search included permutations of the following keywords: wetland plants, marsh plant, bog plant, isoetid, aquatic plants, macrophytes, submerged plants, floating-leaved plants, emergent plants, mangroves, leaf economics traits, leaf economics spectrum, leaf nitrogen, leaf phosphorus, SLA, LMA, leaf life span, photosynthetic rate, underwater photosynthetic rate, dark respiration rate. Additionally, our network of wetland experts from around the world contributed recommendations for possible literature that we had not retrieved. Finally, we added unpublished data of our own and of our network. In combination, we aimed to be as complete as possible without predetermined sample size. Any constraint because of the sample size obtained were accounted for in the statistical analysis.
Data collection	The database has been compiled over a period of 10 years, starting with dedicated workshops (two), followed by continued additions of papers retrieved from literature by the last author. Over the past three years, the first author executed a literature search, finalised the database and run various data quality checks (e.g. on units, outliers, range of values and trait-trait correlations)
Timing and spatial scale	The data in the database refer to publications of which the oldest are from the 1960s and the most recent from publications from the year 2018. We did not restrict our search to specific time periods, as we considered the observed trait values to be representative of the plant species involved. Data are meant to represent global patterns, with most data coming from Europe, United states, China and Australia/New Zealand.
Data exclusions	For the meta-analysis, we excluded plant species with Ellenberg moisture value< 7 to represent strict wetland plants. If data quality checks suggested outliers, the original entries from the papers were checked and if necessary the author of the publication was consulted. This exclusion criteria were pre-established before data analyses.
Reproducibility	The data analysed in this study are available at DRYAD (https://doi.org/10.5061/dryad.v6wwpzgsq) and GLOPNET database (https://www.nature.com/articles/nature02403#Sec15). The R software code can be downloaded from Supplementary Information. People can reproduce the results by re-analysing the original data based on the code provided.
Randomization	The analyses were conducted on all the available wetland plant trait data. In this study, no data randomization procedure was applied in order to keep the largest dataset as possible.
Blinding	Blinding was not possible, as all data have been derived from published literature.

Did the study involve field work? ☐ Yes ☒ No

Reporting for specific materials, systems and methods

We require information from authors about some types of materials, experimental systems and methods used in many studies. Here, indicate whether each material, system or method listed is relevant to your study. If you are not sure if a list item applies to your research, read the appropriate section before selecting a response.

Materials & experimental systems

n/a	Involved in the study
☒	☐ Antibodies
☒	☐ Eukaryotic cell lines
☒	☐ Palaeontology and archaeology
☒	☐ Animals and other organisms
☒	☐ Human research participants
☒	☐ Clinical data
☒	☐ Dual use research of concern

Methods

n/a	Involved in the study
☒	☐ ChIP-seq
☒	☐ Flow cytometry
☒	☐ MRI-based neuroimaging
